# New Delhi metallo-β-lactamase–producing *Escherichia coli* among dogs at an animal rescue facility—Wisconsin, 2022

**DOI:** 10.1017/ash.2023.354

**Published:** 2023-09-29

**Authors:** Kiara McNamara, Caroline Habrun, W. Wyatt Wilson, Leslie Kollmann, G. Sean Stapleton, Richard Stanton, Katharine Benedict, Amanda Beaudoin, Paula Snippes, Melissa Anacker, Megin Nichols, Maroya Walters, Jordan Mason, Nikki Mueller

## Abstract

**Background:** New Delhi Metallo-β-lactamase (NDM)–producing *Escherichia coli* are highly resistant organisms that spread quickly. In the United States, organisms with *bla*NDM are rare and mostly associated with healthcare settings. However, in other countries, *bla*NDM can be relatively common and are found in community settings. State veterinary and public health partners detected NDM *E. coli* in a dog from Iran living at a Wisconsin animal rescue facility (ARF), where 40% of dogs had international origins. We investigated to determine spread among dog and human contacts and prevent further transmission. **Methods:** We screened dogs and humans at the ARF, a local veterinary clinic (clinic A), and ARF staff homes (homes A and B) for colonization with *bla*NDM. We reviewed veterinary records and conducted a case–control analysis to identify risk factors for *bla*NDM acquisition among dogs. We evaluated ARF infection control practices. Screening specimens that were positive for *bla*NDM were cultured. We conducted an analysis of short- and long-read whole-genome sequencing data to evaluate isolate relatedness. We compared NDM *E. coli* sequences from dogs to all NDM *E. coli* sequences from humans collected in Wisconsin and nearby states. **Results:** Screening identified *bla*NDM colonization in 27 (37%) of 73 ARF dogs and 4 (56%) of 7 dogs in home A, but not in ARF or staff in clinic A. Among ARF dogs with *bla*NDM, 20 (74%) 27 had international origins and 22 (81%) had ≥1 medical condition. Dogs sharing the same space (OR, 5.1; 95% CI, 1.8–14.7) were associated with *bla*NDM acquisition. We observed high animal density, soiled environments, and insufficient hand hygiene. ARF staff wore workwear and work shoes off site, including to home A. Sequencing identified 3 multilocus sequence types (STs) using the Achtman scheme among 27 isolates with *bla*NDM-5. Most isolates were ST361 (20 of 27, 74%) followed by ST167 (6 of 27, 22%) and ST1163 (1 of 27, 4%). Within-MLST cluster variability was <1–3 high-quality single-nucleotide variant differences, each harboring a ST-specific plasmid with blaNDM-5. No NDM-*E. coli* sequences from humans appeared related. **Conclusions:** Investigation of a single isolate led to identification of widespread NDM-*E. coli* transmission among dogs at an ARF. There were multiple NDM *E. coli* introductions to the ARF, likely by dogs of international origin. Poor hygiene contributed to transmission among ARF dogs and to dogs outside the ARF. Transmission of *bla*NDM-5 at the ARF and offsite spread to home A demonstrate the potential for unrecognized community sources to disseminate NDM *E. coli* in community settings. Strategies and lessons learned from interventions to prevent antibiotic resistance in human healthcare settings may inform and support prevention in animal care.

**Disclosures:** None

